# Obesity and Dietary Added Sugar Interact to Affect Postprandial GLP-1 and Its Relationship to Striatal Responses to Food Cues and Feeding Behavior

**DOI:** 10.3389/fendo.2021.638504

**Published:** 2021-03-31

**Authors:** Sabrina Jones, Shan Luo, Hilary M. Dorton, Alexandra G. Yunker, Brendan Angelo, Alexis Defendis, John R. Monterosso, Kathleen A. Page

**Affiliations:** ^1^ Division of Endocrinology, Department of Medicine, Keck School of Medicine, University of Southern California, Los Angeles, CA, United States; ^2^ Keck School of Medicine, Diabetes and Obesity Research Institute, University of Southern California, Los Angeles, CA, United States; ^3^ Department of Psychology, University of Southern California, Los Angeles, CA, United States; ^4^ Neuroscience Graduate Program, University of Southern California, Los Angeles, CA, United States

**Keywords:** striatum, glucagon-like peptide-1, obesity, dietary sugar, fMRI, feeding behavior, appetite and food intake

## Abstract

It has been hypothesized that the incretin hormone, glucagon-like peptide-1 (GLP-1), decreases overeating by influencing mesolimbic brain regions that process food-cues, including the dorsal striatum. We previously showed that habitual added sugar intake was associated with lower glucose-induced circulating GLP-1 and a greater striatal response to high calorie food cues in lean individuals. Less is known about how dietary added sugar and obesity may interact to affect postprandial GLP-1 and its relationship to striatal responses to food cues and feeding behavior. The current study aimed to expand upon previous research by assessing how circulating GLP-1 and striatal food cue reactivity are affected by acute glucose consumption in participants with varied BMIs and amounts of habitual consumption of added sugar. This analysis included 72 participants from the Brain Response to Sugar Study who completed two study visits where they consumed either plain water or 75g glucose dissolved in water (order randomized; both drinks were flavored with non-caloric cherry flavoring) and underwent repeated blood sampling, a functional magnetic resonance imaging (fMRI) based food-cue task, and an ad-libitum buffet meal. Correlations between circulating GLP-1 levels, striatal food-cue reactivity, and food intake were assessed, and interactions between obesity and added sugar on GLP-1 and striatal responses were examined. An interaction between BMI and dietary added sugar was associated with reduced post-glucose GLP-1 secretion. Participants who were obese and consumed high levels of added sugar had the smallest increase in plasma GLP-1 levels. Glucose-induced GLP-1 secretion was correlated with lower dorsal striatal reactivity to high-calorie versus low-calorie food-cues, driven by an increase in reactivity to low calorie food-cues. The increase in dorsal striatal reactivity to low calorie food-cues was negatively correlated with sugar consumed at the buffet. These findings suggest that an interaction between obesity and dietary added sugar intake is associated with additive reductions in postprandial GLP-1 secretion. Additionally, the results suggest that changes to dorsal striatal food cue reactivity through a combination of dietary added sugar and obesity may affect food consumption.

## Introduction

Obesity among U.S. adults has reached over 40% of the total population (1). In order to combat this public health crisis, recent research has been aimed at identifying interactions between neural and hormonal mechanisms that underly energy regulation and the factors that can disrupt typical functioning, inducing a cycle of overeating and excess weight gain. One hormone that has been identified as a key influence in the control of consummatory behavior is glucagon-like peptide-1 (GLP-1). GLP-1 is an incretin hormone that is derived from preproglucagon and is predominantly produced in intestinal L-cells (2) and a subset of neurons in the nucleus tractus solitarius (NTS) (3). Endogenous GLP-1 is elevated following glucose consumption (4) and improves glucose metabolism by augmenting glucose stimulated insulin secretion (5) and decreasing glucagon levels (4).

Along with its effects on glucose metabolism, GLP-1 has been implicated in altering the brain’s processing of both food and drug rewards (6) through its influence on the mesolimbic system (7–9). Activation of GLP-1 receptors using GLP-1 analogues decreases drug reward and striatal c-fos expression in mice (10). In humans, neuroimaging studies in humans have found that infusions of GLP-1 (11) and GLP-1 agonists (12), or glucose-induced GLP-1 increases (13, 14) alter brain responses to food cues in regions of the brain involved in the regulation of eating. A previous study from our laboratory (15) found a negative correlation between GLP-1 response to oral glucose and activation of the dorsal striatum in response to high-calorie food images, relative to nonfood images, in lean young adults.

In addition, this study showed that high levels of habitual added sugar consumption were associated with both a reduction in GLP-1 secretion in response to acute glucose ingestion and an increase in dorsal striatal food cue reactivity to palatable food cues. While this study provided new insights into relationships between consumption of dietary added sugars, GLP-1 secretion, and striatal food cue reactivity, these relationships were only examined in lean participants and the food cue task only included high-calorie food cues (15). Excess weight gain (e.g., obesity/overweight) has been associated with impairments in glucose-induced GLP-1 secretion (16–19) and alterations to dorsal striatal response to food cues (20–23), but no study that we are aware of has examined how an interactions between obesity and dietary added sugars are related to postprandial GLP-1 secretion or dorsal striatal food cue reactivity, despite the established potential comorbidity between these two factors (24, 25).

The current study aimed to extend findings from Dorton et al. (15) by examining if dietary added sugar interacts with overweight and/or obesity to alter glucose-induced GLP-1 and striatal food cue reactivity. Additionally, the current study assessed if these relationships were specific to high-calorie, palatable food cues, like those used in Dorton et al. (15), or if they applied to low-calorie food cues. Finally, the current study examined if the observed relationships were associated with changes to consummatory behavior. Data was collected as part of a larger randomized cross-over study in which neuroimaging, blood sampling, and an ad-libitum buffet meal were performed to examine the neuroendocrine regulation of feeding behavior. Based on our previous findings, we expected post-glucose GLP-1 levels to be correlated with striatal food cue reactivity, but we predicted that the direction of this relationship could be food cue type specific as the striatum has been found to respond differentially to high calorie and low calorie food cues based on interoceptive state (26). We hypothesized that BMI, dietary added sugar, and/or the interaction between the two would be associated with reductions in postprandial GLP-1 secretion and increased striatal food cue reactivity. The behavioral relevance of dorsal striatal and/or GLP-1 alterations was also tested by correlating the neuroimaging and hormone data with food consumption at an ad-libitum buffet meal.

## Subjects and Methods

### Participants

All participants from the parent study that completed either the water or glucose days were included in the present analysis. Using this inclusion criteria, data on 72 young adults (32 males, 40 females) were included in this analysis. Participants were right-handed, nonsmokers, weight stable for at least 3 months, non-dieters, not on any medication (except oral contraceptives), with normal or corrected-to-normal vision, and no history of diabetes, eating disorders, or other significant medical diagnoses. Recruitment occurred between July 2016 and January 2020. During the course of the study, participants were asked to adhere to their usual diet and physical activity levels. Participants provided written informed consent compliant in accordance with the Declaration of Helsinki. The protocol # HS‐09‐00395‐CR011 was approved by the University of Southern California Institutional Review Board.

Three participants were excluded from the final analysis of dorsal striatal food cue reactivity on the glucose day: one because of ≥5% weight gain between the water and glucose sessions, one for scanner sequence error during the food cue task, and one for motion (larger than 2 mm or 2° in any direction). Four participants were excluded from analysis of food cue reactivity on the water day: one because of drop out, one because of scanner failure during data collection for the food cue task, and two because of motion. Sixty-nine participants were included in the final glucose food cue reactivity analysis and sixty-eight were included in the final water food cue reactivity analysis. Sixty-six of these participants completed blood sampling on the glucose day and sixty-two completed blood draws on the water day. Problems with intravenous line insertion or blood sampling led to the lower numbers of participants who completed blood sampling procedures (for participant flowsheet for this sub-study see **Supplemental Figure 1**). There were no significant differences in demographics of the included vs excluded participants (see **Supplemental Results**).

### Experiment Overview

This study used data collected as part of a larger study aimed at examining the neural mechanisms for appetitive responses to food (Clinical Trial NCT02945475). In the larger parent study, each participant attended an initial screening visit and up to four functional magnetic resonance imaging (fMRI) test sessions. This analysis only included two of these fMRI sessions (i.e., water and glucose drinks) to specifically examine how obesity and dietary sugar affect the GLP-1 response to glucose and dorsal striatal responses to food cues. At the screening visit, demographic information was collected along with height (cm), weight (kg), and a 24h dietary intake recall. Height was measured to the nearest 0.1cm using a stadiometer and weight was measured to the nearest 0.1 kg using a calibrated digital scale (Model no.SC-331S, TANITA Corporation of America, Inc.). Using the anthropometric measures acquired at the screening visit, BMI was calculated as weight (kg)/height(m)^2^. At each fMRI study session, height and weight were collected to confirm participants were weight stable (body weight change <5%). Additionally, a 24h dietary intake recall was collected along with fMRI scans, plasma GLP-1 levels, and calories consumed at a buffet. All females underwent study session days during the follicular phase of the menstrual cycle to control for potential cofounding effects of cycle on appetitive behaviors (27, 28).

**Figure 1:**
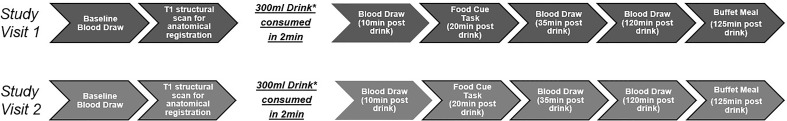
Visualization of the study visits. *300ml noncaloric cherry flavored drink. Either water or 75g of glucose dissolved in water. Order of drinks were counterbalanced.

MRI scans were performed at the Dornsife Cognitive Neuroimaging Center of the University of Southern California. For each test session day, participants arrived at approximately 8:00 a.m. after a 12h overnight fast. Upon arrival, participants were asked to complete a 24h dietary recall and a baseline blood draw was performed. The MRI scan began with a T1 structural scan (used for anatomical registration). Participants then received one of two standard drinks to consume within 2 minutes. One drink was a 75g glucose load dissolved in 300ml of water along with.45g of non-sweetened, zero calorie, cherry flavoring and the other drink was a noncaloric 300ml of water with.45g of non-sweetened, zero calorie, cherry flavoring [drinks based on (15)]. The order of the drink days was randomized for each individual using a computer-generated sequence and the time interval between the two drinks days ranged from 2 days-2 months. Both participants and experimenters were blinded to the drink provided during the study sessions. Following drink consumption, a second blood draw was performed (10min post-drink), participants returned to the scanner for the food cue task (20min post-drink). Blood draws were performed again at 35min and 120 min post-drink. The study ended with a food buffet (125min post-drink) (for study sessions overview see **Figure 1**).

### 24h Dietary Recall

To assess dietary intake, we used the validated multiple-pass 24h dietary recall (29, 30). A trained staff member interviewed participants on all food and beverage items consumed during the prior 24h period. Participants were asked to provide the amount of each item consumed, time of consumption, how each item was prepared, and additional details (e.g., brand name). Recall interviews typically lasted between 30-60min. Dietary recalls were performed during the screening visit and each MRI visit. Data from dietary recalls were manually checked for quality. To determine outliers, we performed a linear regression analysis, using body weight to predict caloric intake. Residuals were standardized and examined for any values that were >3 SDs from the mean (15). Using this method, 359 dietary recalls were included in this analysis (an average of 5 recalls per participant), and 5 recalls were excluded. Participant’s dietary data was averaged across all available recall days.

Dietary data was analyzed using Nutrition Data Systems for Research (NDSR) software version 2015, developed by the Nutrition Coordinating Center, University of Minnesota, Minneapolis, MN, USA. The output from the software provided intake of overall calories and the breakdown of macronutrients. For the purpose of this study, we analyzed the amount of added sugar in the diet as a percent of total calories. Based on the World Health Organization’s dietary recommendations, ≥10% added sugar was considered a high amount of added sugar and <10% was considered a low amount of added sugar based.

### Food-Cue Task

Participants completed the food-cue task in the MRI scanner by viewing stimuli through a mirror mounted over the head coil. In a randomized block design, participants were asked to watch a total of 12 visual food cue and non-food cue blocks using Matlab (MathWorks, Inc., Natick, MA, USA) and Psychtoolbox on a 13-in, 2.5 GHz Intel Core i5 processor MacBook Pro. Four images per block were presented in random order, each appearing immediately after the last. Within a block, each image was presented for 4 s. An 8s questioning period followed each block where participants were asked to rate their hunger along with their wanting and liking for the food cues; however, these data were not included in this analysis. There were different food cue types presented: 4 high-calorie (e.g., pizza, ice cream) food image blocks, 4 low-calorie (e.g., carrots, apples) food image blocks, and 4 non-food (buses, staircase) image blocks (for a full list of visual cues see **Supplemental Table 1**). The set of food and non-food cue images was gathered from the food-pics database (31) and prior published work (15). The total running time of this task was ~6 min.

### Hormone Analysis

Blood samples were assessed for GLP-1 (7–35) (active) using Luminex multiplex technology (Millipore, Billerica, MA). Circulating insulin and glucose were also assessed as both would be expected to be altered, along with GLP-1, by acute glucose ingestion. Plasma glucose was measured enzymatically using glucose oxidase (YSI 2300 STAT PLUS Enzymatic Electrode-YSI analyzer, Yellow Springs Instruments), and plasma insulin was measured *via* Luminex multiplex technology (Millipore, Billerica, MA) Insulin and blood glucose levels were controlled for in the data analysis to examine if any observed relationships were GLP-1-dependent or due to confounding hormones. Plasma glucose and hormones were assessed at each time point (baseline/0 min and 10, 35, 120 min post-drink) and area under the curve (AUC) was calculated using the trapezoid method (32).

### MRI Imaging Parameters and Analysis

Imaging data were collected using a 3T Siemens MAGNETOM Prismafit MRI System, with a 32-channel head coil. A high-resolution 3D magnetization prepared rapid gradient echo sequence (TR=1950ms; TE=2.26ms; bandwidth=200Hz/pixel; flip angle=9°; slice thickness=1mm; FOV=224mm×256mm; matrix=224×256) was used to acquire structural images for multi-subject registration. Food cue reactivity was measured by functional BOLD signals, acquired with a multi-band interleaved gradient echo planar imaging sequence. Eighty-eight 1.5-mm thick slices covering the whole brain were acquired using the following parameters: repetition time (TR)=1,000ms, echo time (TE)=43.20ms, bandwidth=2,055Hz/pixel, flip angle=52°, field of view (FOV)=128mm×112mm, matrix=128×112.

To analyze the fMRI data, we used tools from the Oxford University Centre for Functional MRI of the Brain Software Library (FMRIB). MRI data were processed using the fMRI Expert Analysis Tool (FEAT) version 6.00. Eight functional volumes (8 TRs) acquired at the beginning of each MRI session were discarded to account for magnetic saturation effects. fMRI data were preprocessed using motion correction, high-pass filtering (100s), and spatial smoothing with a Gaussian kernel of full width at half-maximum = 5 mm. Functional data were first mapped to each participant’s anatomical image and then registered into standard space [Montreal Neurological Institute (MNI)] using affine transformation with FMRIB’s Linear Image Registration Tool to the avg152 T1 MNI template. Explanatory variables were added to the general linear model after convolution with a canonical hemodynamic response function. Temporal derivatives and temporal filtering were added to increase statistical sensitivity. Motion confounds were generated using the tool “fsl_motion_outliers” to be used as regressors of no-interest in the general linear model. For each participant, contrast maps were created on the first-level analysis for: high-calorie food vs nonfood, low-calorie food vs nonfood, and high-calorie food vs low-calorie food images.

To specifically test the relationship between postprandial GLP-1, BMI, dietary added sugar, food consumption and the dorsal striatum food cue reactivity, we used an *a priori* ROI-based approach. An anatomical, bilateral ROI of the striatum (including caudate and putamen) was created using the Harvard-Oxford subcortical atlas with a probability threshold over 50% (**Supplemental Figure 2**). Percent signal change was extracted from the striatal ROI for contrasts for each participant using FSL’s FEATquery.

Additional arterial spin labeling scans were conducted pre-drink and 5 and 26min post-drink, but this data was not analyzed in the current sub-study.

### Buffet Meal

Study sessions ended with the presentation of an ad libitum buffet meal given 125min post-drink. The buffet meal consisted of 32 pre-measured food and drink items, including high-calorie foods, such as potato chips and cookies, and lower calorie foods, such as apple slices and carrots. Total energy available from the buffet meal was 4650kcal (for a full list of foods at the buffet and the calorie content of each food cue see **Supplemental Table 2**). Caloric value per gram or fluid ounce of each item was calculated using the NDSR. Participants were given 20 minutes to eat any quantity they desired and instructed not to leave the room with any items. After the participant exited, each buffet item was re-weighed. Calorie and nutrient intake during the buffet meal were calculated using the difference between the pre-meal and post-meal weight for each buffet item.

### Statistical Analysis

All statistical tests were corrected for age and gender. Alpha levels were set at.05 for all tests, except were alpha was adjusted using Bonferroni corrections, and all confidence intervals (CI) were set at 95%. Partial η^2^ were used to calculate effect size. Statistical analyses were run using Statistica Academic 13.3.0 (Tibco).

#### Relationship Between BMI, Dietary Added Sugar and GLP-1 or Striatal Food Cue Reactivity

Simple regressions were run using BMI or percent calories from added sugar as the regressor and GLP-1 AUC (pg/ml) or striatal food cue reactivity (% signal change) as the dependent variables. Factorial regressions were then run to examine interactions between BMI and dietary added sugar on striatal food cue reactivity. The striatal food cue reactivity (measured as percent signal change) contrasts assessed were: high-calorie food vs nonfood, low-calorie food vs nonfood, and high-calorie food vs low-calorie food images. These tests were run on striatal food cue reactivity separately for water and glucose days. Findings were controlled for age and gender.

#### Correlations Between GLP-1 and Striatal Food Cue Reactivity

Multiple regression analyses were run using GLP-1 AUC on water or glucose days as the primary regressor, and BMI, percent calories from added sugar, age, and gender as co-variate regressors and striatal food cue reactivity (following water or glucose) as the dependent variable. Findings were further corrected for potentially confounding hormones that are also altered by glucose consumption (plasma insulin and circulating glucose AUC) and differences in insulin sensitivity [calculated as the Matsuda Index (33)]. All striatal contrasts listed above were tested.

#### Striatal Food Cue Reactivity and Consumption at a Buffet

Paired t-tests were run to assess if differences in buffet consumption on water and glucose days. Simple regressions were run using BMI or percent calories from added sugar as the regressor and consumption at a food buffet at the dependent variables. Buffet consumption measures that were tested included total calories consumed and calories consumed of macronutrients (sugar, fat, protein, total carbohydrates). Factorial regressions were also run to test for an interaction between BMI and dietary added sugar on food consumption. To assess the role of circulating GLP-1 and striatal food cue reactivity multiple regressions were used. Striatal food cue reactivity contrasts found to significantly correlate with plasma GLP-1 following glucose consumption, GLP-1 AUC, BMI, percent calories from added sugar, age, and gender were co-variate regressors and consumption measures as the dependent variable.

## Results

### Participants

Mean Age (23.22 ± 3.73years), Body mass index (BMI) (27.33 ± 5.13 kg/m^2^), and Consumption of Dietary Added Sugar (9.31 ± 4.47%) for the overall cohort of 72 (32 males, 40 females) participants are described in **Table 1**. Independent samples t-tests found no significant differences in age, BMI, or percent calories from added sugar in the participants that completed all measurements (food cue task on the glucose and water days and hormone on glucose and water days) and the participants that either did not completed all assays or were removed from data analysis for confound, like motion. For detailed t-test findings and descriptive statistics stratified by BMI and Added Sugar see **Supplemental Results**.

**Table 1:** Characteristics for all participants (N=72).

CHARACTERISTIC	Mean ± SD
*SEX*	Male: n=32
	Female: n=40
*AGE (YEARS)*	23.22 ± 3.73
*BMI (KGIM2*)	27.33 ± 5.13
* LEAN (>18.5 AND <24.9)*	n=25; 22.13 ± 1.67
* OVERWEIGHT (>25.0AND <29.9)*	n=25; 26.93 ± 1.28
* OBESE (>30.0)*	n=22; 33.68 ± 3.05
*PERCENT CALORIES FROM ADDED SUGAR*	9.31 ± 4.47
* LOW (<10%)*	n=42; 6.59 ± 2.38
* HIGH (>=10%)*	n=30; 13.16 ± 3.88

### GLP-1 Following Drink

Across all participants, paired t-tests showed that circulating GLP-1 levels significantly increased 10 min (t(65)=6.26, 95%CI [8.01, 15.5], p<.001), 35 (t(65)=6.55, 95%CI:[5.71, 10.72], p<.001), and 120 min (t(65)=4.72, CI[2.57, 6.34], p<.001) post-glucose drink relative to pre-drink. Conversely, GLP-1 levels significantly decreased 10 min (t(61)=-2.69, 95%CI[-1.8, -.26], p<.01), 35 (t(61)=-3.98, 95%CI[-2.8, -.93], p<.001), and 120 min (t(61)=-2.69, 95%CI[-2.51, -.37], p<.01) post-water drink relative to pre-drink. T-tests also revealed that GLP-1 AUC was significantly greater following a glucose versus water drink (t(58)=9.65, 95%CI[1179.9, 774.414], p<.001).

### Relationship Between BMI and Dietary Added Sugar on Circulating GLP-1

**Figure 2:**
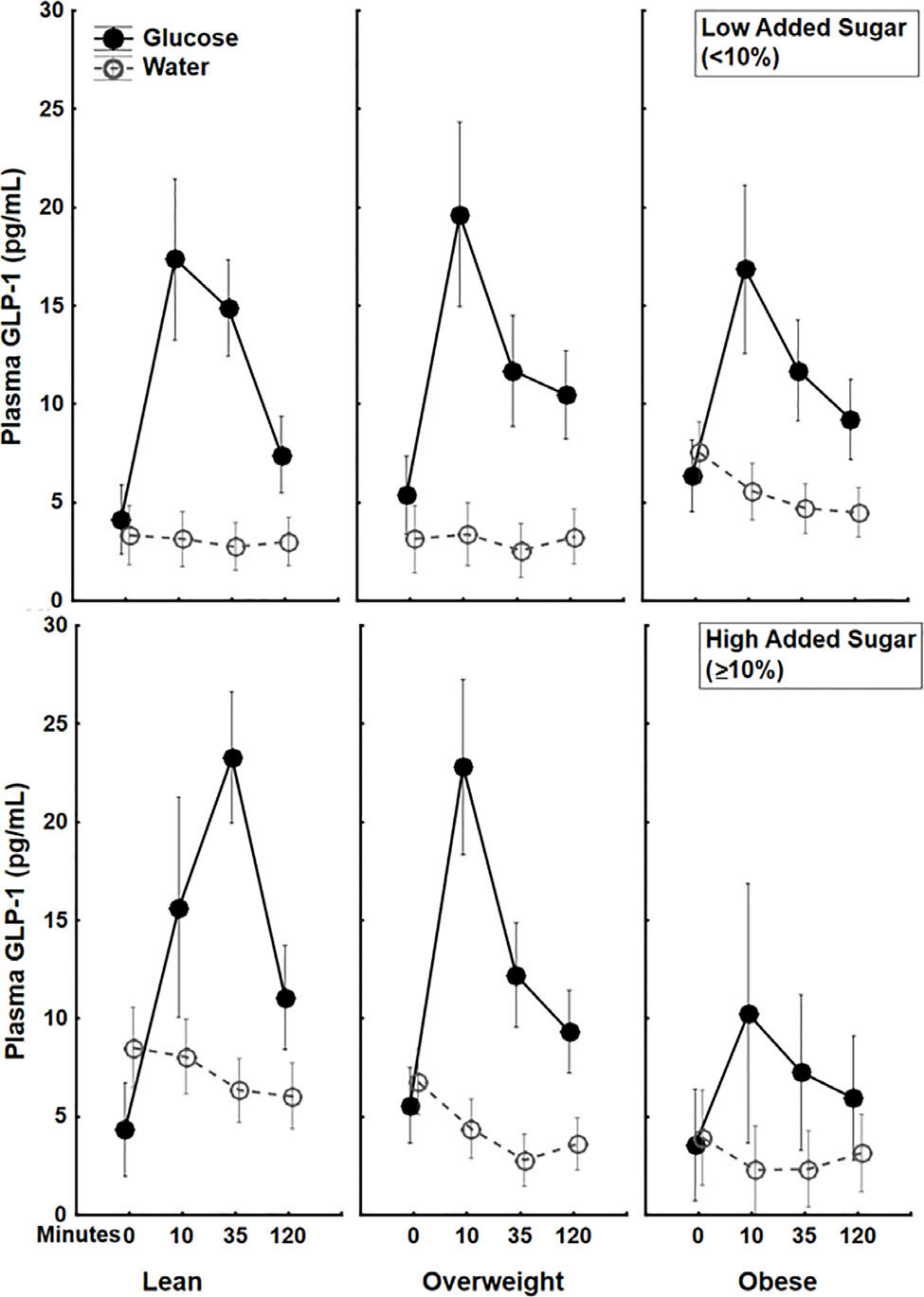
Plasma 5(pg/mL) trajectories in response to glucose (75 g) (closed circles) or water (open circles) drink stratified by both BMI status and Dietary Added Sugar intake. The additive effect of BMI and dietary added sugar are particularly apparent among individuals with obesity who consumed high levels of dietary added sugar (≥10%)*. *The World Health Organization and the US Department of Health and Human Services recommended that daily intake of added sugars should account for less than 10% of the total calories consumed (36).

Simple regressions showed a trend towards a negative correlation between BMI and circulating GLP-1 (AUC) (β=-.21, p=.08), but no significant correlation between percent calories from added sugar and circulating GLP-1 (AUC) (β=.09, p=.49) following a glucose drink. Interestingly, a factorial ANOVA examining the interaction between BMI and dietary added sugar showed a significant BMI × percent calories from added sugar interaction (β=-1.87 ± .85, 95%CI[-3.56, -.18], p<.05, partial η^2^=.07) on circulating GLP-1 (AUC) following the oral glucose load. The increase in GLP-1 secretion following glucose was markedly lower among participants with both high BMI and high percent calories from added sugar in their diet (see **Figure 2**). There were no significant correlations between BMI (p=.41) or percent calories from added sugar (p=.8) as well as no significant interaction between BMI and percent calories from added sugar on circulating GLP-1 following water ingestion (p=.76) (**Figure 2**). These findings are the first to illustrate that the interaction between BMI and percent calories from added sugar is associated with decreased GLP-1 secretion following glucose ingestion. The interaction of BMI and dietary added sugar on GLP-1 secretion demonstrated that individuals with obesity who consumed higher levels of dietary added sugar had the lowest postprandial GLP-1 response.

### Associations Between BMI and Dietary Added Sugar on Striatal Food Cue Reactivity Following Glucose or Water

Simple regressions showed no correlations between BMI (all p>.15) or percent calories from added sugar (all p>.1) and striatal food cue reactivity following glucose or water consumption. Additionally, a factorial regression examining the interaction between BMI and percent calories from added sugar yielded no significantly correlations between the interaction term and striatal food cue reactivity to any food cue type following glucose or water (all p>.2).

### GLP-1 and Striatal Food Cue Reactivity

**Figure 3:**
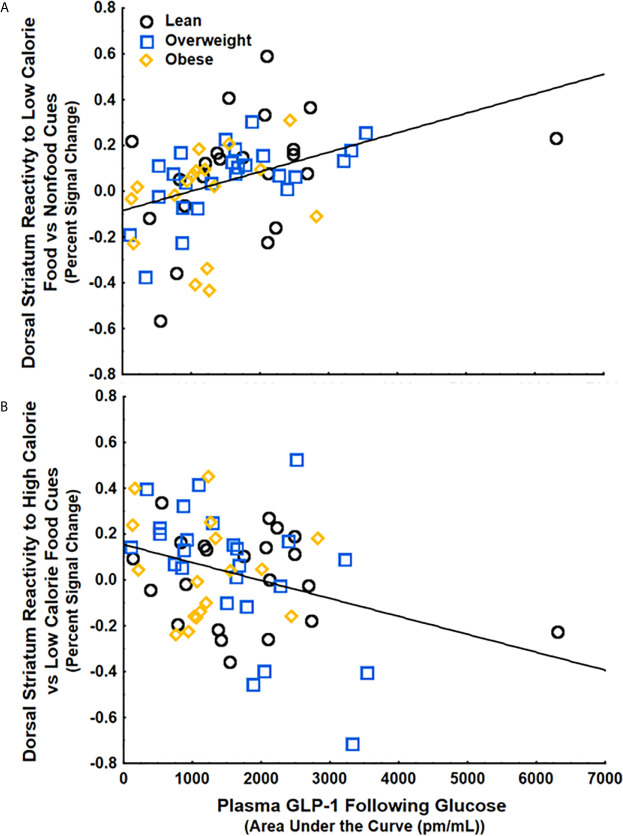
GLP-1 secretion following glucose ingestion was **(A)** positively correlated with striatal food cue reactivity to low-calorie food vs nonfood cues (p<.01) and **(B)** negatively correlated with striatal food cue reactivity to high-calorie relative to low calorie food cues (p<.05). These findings were controlled for BMI, percent calories from added sugar, age, and gender.

To examine if circulating GLP-1 is related to food cue processing, we ran a multiple regression with GLP-1, BMI, and percent calories from added sugar as regressors and striatal food cue reactivity following either water or glucose as the dependent variable. We found that GLP-1 secretion following glucose, but not BMI or Percent Calories from Added Sugar, was positively correlated with striatal food cue reactivity to low-calorie food cues vs non-food cues (β=.4 ± .12, 95%CI[.15,.64], p<.01, partial η^2^=.15) (**Figure 3A**) and negatively correlated with striatal food cue reactivity to high-calorie relative to low-calorie food cues (β=-.37 ± .13, 95%CI[-.62, -.11], p<.01, partial η^2^=.13) (**Figure 3B**). These results were adjusted for age and gender, which also did not have significant correlations with striatal food cue reactivity. There were no correlations between GLP-1, BMI and/or percent calories from added sugar and striatal food cue reactivity after water consumption. The positive correlation between GLP-1 and dorsal striatal responding to low calorie food cues relative to nonfood cues (β=.49 ± .14, 95%CI[.15,.68], p<.01, partial η^2^=.15) and the negative correlation between GLP-1 and dorsal striatal responding to high calorie food cues relative to low calorie food cues (β=-.40 ± .14, 95%CI[-.67, -.11], p<.01, partial η^2^=.13) remained significant after further adjusting for changes in circulating insulin and glucose levels in response to glucose ingestion and further adjusting for insulin sensitivity. Additionally, these findings remained significant after adjusting the alpha level using Bonferroni corrections to account for repeated measures (alpha=.016). Taken together, the data suggest that following glucose consumption, increases in circulating GLP-1 are associated with an increased preferential response to low- vs high-calorie foods driven by increased reactivity to low-calorie food cues.

### Consummatory Behaviors

Overall caloric consumption at the buffet was greater on water relative to glucose days (t(68)=3.94, 95%CI[63.61, 194.01],p<.001), and consumption of all macronutrients was greater on water vs. glucose days (all p<.05).

BMI and percent calories from added sugar were not independently correlated with any food consumption measures on water or glucose days (all ps>.2), and there was no interaction between BMI and percent calories from added sugar on food consumption at the buffet on water or glucose days (all ps>.3).

**Figure 4:**
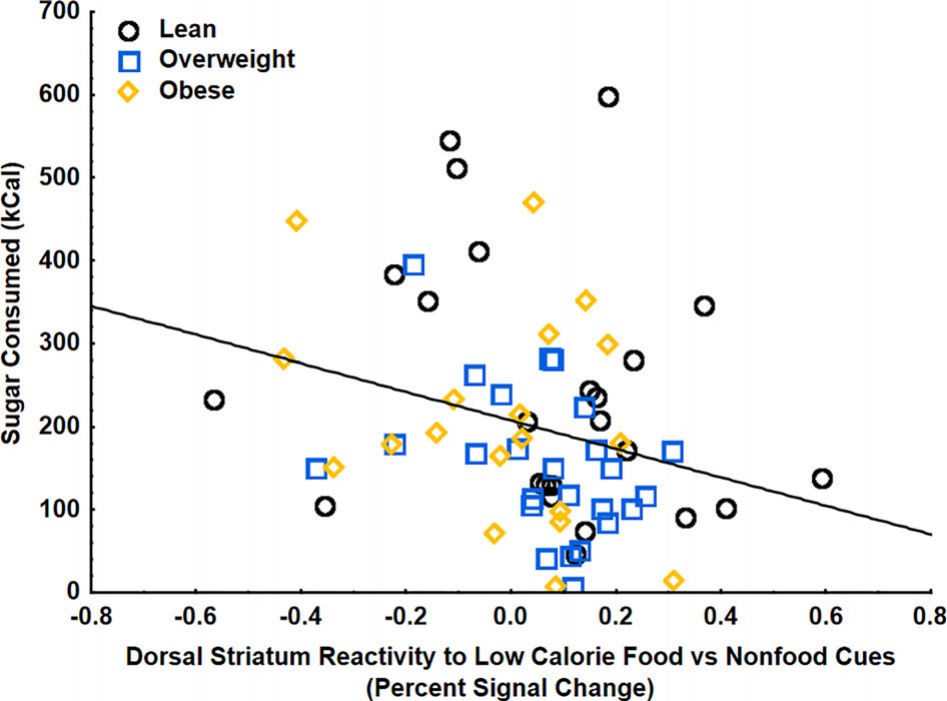
Striatal food cue reactivity to low calorie food vs nonfood cues was negatively correlated with sugar (kcal) consumed at the food buffet on the glucose day (p<.05). These findings were controlled for age, gender, BMI, percent calories from added sugar, and glucose-induced increases in GLP-1.

Using multiple regression analyses, striatal food cue reactivity to low-calorie food cues (vs. non-food cues) was negatively correlated with sugar (kcal) consumed at the food buffet following a glucose drink, even when adjusting for age, gender, BMI, percent calories from added sugar, and postprandial GLP-1 (β=-.32 ± .14, 95%CI[-.59, -.05], p<.05, partial η^2^=.09) (**Figure 4**). There were no significant relationships between BMI, percent calories from added sugar, post-prandial GLP-1 levels, or interactions between these variables with consumption of sugar at the buffet, indicating that changes in striatal food cue reactivity, rather than body weight, dietary added sugars, or circulating GLP-1, may be driving the changes in consummatory behaviors. There were no correlations between striatal food cue reactivity to low-calorie food cues and total carbohydrates, fat, protein, or total calories consumed. There were also no correlations between striatal reactivity to high vs low calorie food cues, GLP-1, BMI, or percent calories from added sugar and total caloric intake at the buffet after glucose consumption. There were also no correlations between striatal food cue reactivity, GLP-1, BMI, percent calories from added sugar, and food consumption at the buffet after consumption of water (control). These findings suggest a potential shift of striatal food cue reactivity from high-calories food cues to low-calorie food cues after glucose consumption and this shift is related to a reduction in ad-libitum sugar intake.

## Discussion

The current paper supports a potential indirect pathway by which GLP-1 regulates appetitive behaviors through its impacts on dorsal striatal food cue reactivity. Furthermore, the present findings help to describe how the interaction between BMI and dietary added sugars may be associated with disruptions in this mechanism. To our knowledge this is the first study to illustrate that an interaction between BMI and dietary added sugar intake is associated with altered postprandial GLP-1 secretion. Post-glucose circulating GLP-1 levels were positively correlated with dorsal striatal food cue reactivity to low-calorie food vs nonfood cues and negatively correlated with dorsal striatal food cue reactivity to high-calorie vs low-calorie food cues, independent of BMI and dietary added sugar. The relationship between glucose-induced GLP-1 secretion and dorsal striatum food cue reactivity suggests that increases in plasma GLP-1 following glucose consumption may shift dorsal striatum food cue reactivity from high-calorie food cues toward low-calorie food cues. This potential shift, represented by the increase in dorsal striatal response to low-calorie food cues, was associated with a decrease in sugar intake at the buffet meal. These results suggest that postprandial increases in GLP-1 may play an indirect role in regulating eating behaviors through preferential striatal responsivity to low-calorie (healthier) foods. How these findings relate to signaling between other brain regions and networks involved in appetite regulation remains to be elucidated. Future studies should assess how the current findings may be related to other food cue reactivity alterations in other appetite processing regions of the brain.

Taken together, the current study suggests a potential GLP-1 and dorsal striatal mechanism for how dietary added sugar may affect eating behavior in individuals with overweight and obesity, but the correlational nature of these findings prevents the conclusion of directionality. While the present experiment cannot rule out other confounding factors that may be associated with glucose ingestion, the observed association between increases in circulating GLP-1 and dorsal striatal activation to low-calorie food cues was independent of postprandial increases in plasma glucose or insulin levels or insulin sensitivity. However, to address the directionality question and rule out third variable effects, an experimental design assessing the effect of GLP-1 administration or a GLP-1 antagonist on food cue reactivity and eating behavior is necessary.

GLP-1 analogues have been found to be associated with weight loss in clinical populations with obesity and overweight with or without diabetes (34, 35, 37), but individual effectiveness of these weight loss therapies is highly variable. These findings could suggest that GLP-1 analogues, such as liraglutide and exendin-4, may be especially successful weight loss therapies in individuals more likely to exhibit decreases in GLP-1 secretion (e.g., people with obesity and those who habitually consume high levels of dietary added sugar), but this prediction requires further investigation with randomized controlled studies. Identifying at risk populations may help inform individualized treatment strategies and improve the success rate of weight loss and weight loss maintenance interventions.

## Data Availability Statement

Imaging data is available at Open Source Framework (OSF) https://osf.io/E7B9F/. Additional data generated and analyzed during the current study are available from the corresponding author (KP), on reasonable request. Further inquiries can be directed to the corresponding author.

## Ethics Statement

The studies involving human participants were reviewed and approved by # HS‐09‐00395‐CR011 was approved by the University of Southern California Institutional Review Board. The patients/participants provided their written informed consent to participate in this study.

## Author Contributions

SJ, SL, HD, JM, and KP were responsible for conceptualization of the study. SJ and BA contributed to methodology and formal analysis. HD, AY, BA, and AD were responsible for management and coordination of the study execution. KP was responsible for supervision of the research activities. SJ and KP wrote the original draft. SJ, SL, AY, JM, and KP provided critical review, commentary, and revisions to the manuscript. KP provided funding for this study. All authors contributed to the article and approved the submitted version.

## Funding

This work was supported by the National Institutes of Health (NIH) National Institute of Diabetes and Digestive and Kidney Diseases R01DK102794 (PI: KP). A Research Electronic Data Capture, REDCap, database was used for this study, which is supported by the Southern California Clinical and Translational Science Institute (SC CTSI) through NIH UL1TR001855. American Heart Association 14BGIA18720032 (PI: KP).

## Conflict of Interest

The authors declare that the research was conducted in the absence of any commercial or financial relationships that could be construed as a potential conflict of interest.
