# Supplementary material for: Obesity and Dietary Added Sugar Interact to Affect Postprandial GLP-1 and Its Relationship to Striatal Responses to Food Cues and Feeding Behavior
Source: Front Endocrinol (Lausanne). 2021 Mar 31;12:638504. doi: 10.3389/fendo.2021.638504 (PMC8044510; doi:10.3389/fendo.2021.638504)
Supplement: Supplementary file 1 [file DataSheet_1.pdf]

Supplementary Material

Results

Participants

Independent t-tests found no differences in age (completed=23.19 years, not completed=23.95 years, p=.72) BMI(completed=27.19 kg/m^2, not completed=30.36 kg/m^2, p=.29), or percent calories from added sugar (completed=9.26%, not completed=10.14%, p=.74) between participants that completed the glucose food cue task relative to those that did not. Age (completed=23.29 years, not completed=21.85 years, p=.45), BMI(completed=27.4 kg/m^2, not completed=26 kg/m^2, p=.59), and dietary added sugar(completed= 9.47%, not completed=6.52%, p=.2) also did not differ between those that completed the water day food cue task and those that did not. Similarly, age (completed=23.17 years, not completed=23.73 years), BMI (completed=27.29 kg/m^2, not completed=27.76 kg/m^2, p=.82), and percent calories from added sugar (completed=9.28%, not completed=9.62%, p=.85) did not differ between subjects that completed the hormone collection on the glucose day vs those that did not and age (completed=23.1 years, not completed=23.94 years, p=.51), BMI (completed=27.4 kg/m^2, not completed=26.87 kg/m^2, p=.76), and percent calories from assed sugar (completed=9.36%, not competed=8.96%, p=.79) did not differ between participants that completed the hormone collection on the water day and those that did not. Characteristics of each subset of participants can be found in Supplemental Table 3.

Mean age, BMI, and dietary added sugar separated by BMI Status (lean, overweight, and obese) can be found in Supplemental Table 4a. Across weight groups a one-way ANOVA found there was no significant differences in age (F(2,69)=2.15, p=.12) or percent calories from added sugar (F(2,69)=1.7, p=.19). As expected, all three BMI Status groups were significantly different in BMI (F(2,69)=178.58, p<.0001). Descriptive characteristics for High vs Low Added Sugar can be found in Supplemental Table 4b. Independent samples t-tests found High vs Low Added Sugar did not differ in age (Low=23.4±3.89 years, High=22.95±3.54 years, p=.62) or BMI (Low=27.45±5.59 kg/m^2, High=27.15±4.45 kg/m^2, p=.81). The High Added Sugar group had consumed significantly greater percent added sugar than the Low Added Sugar group (Low=6.59±2.38%, High=13.49±3.99%, p<.0001). A factorial ANOVA also found there were no significant interactions between BMI Status and High vs Low Added sugar on BMI (F(2,66)=.28, p=.76) or percent calories from added sugar (F(2,66)=.57, p=.57).

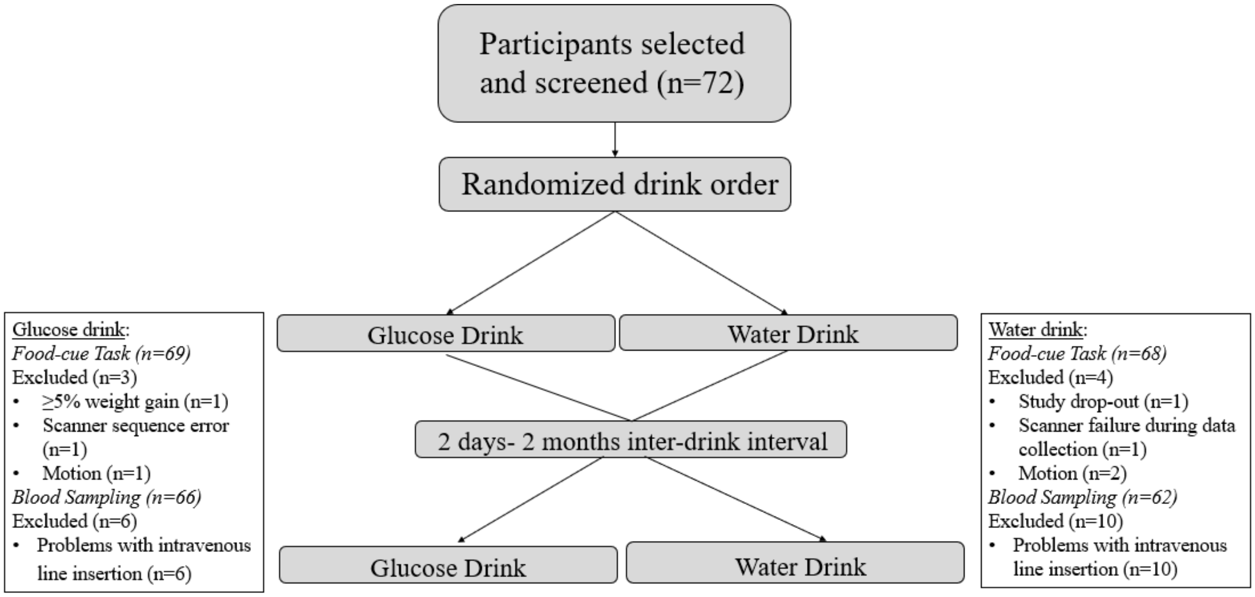

Supplemental Figure 1. Participant flow chart for the current sub-study.

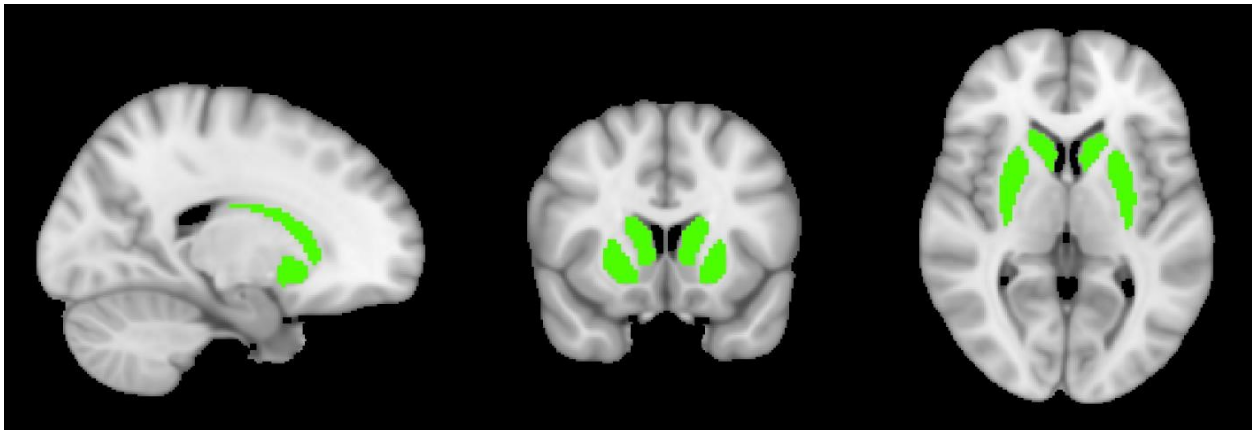

Supplemental Figure 2. Region of interest (ROI) mask of the left and right dorsal striatum derived from the Harvard-Oxford subcortical atlas.

Supplemental Table 1. Visual cues used for the food cue task

Supplemental Table 1: Visual Cues

Food cue	Food cue category
Brownie	High-calorie
Burger	High-calorie
Chocolate bar	High-calorie
Chocolate chip cookie	High-calorie
Cinnamon roll	High-calorie
Cupcake	High-calorie
Donut	High-calorie
Fried chicken	High-calorie
Fries	High-calorie
Gummies	High-calorie
Ice cream	High-calorie
Mac & cheese	High-calorie
M&Ms	High-calorie
Pizza	High-calorie
Potato chips	High-calorie
Taco	High-calorie
Apple	Low-calorie
Banana	Low-calorie
Bell pepper	Low-calorie
Blueberry	Low-calorie
Broccoli	Low-calorie
Carrots	Low-calorie
Chicken salad	Low-calorie
Cucumber	Low-calorie
Fruit salad	Low-calorie
Grape	Low-calorie
Hummus	Low-calorie
Mixed fruit	Low-calorie
Orange	Low-calorie
Popcorn	Low-calorie
Salad	Low-calorie
Salmon	Low-calorie
Basket	Non-food
Bike	Non-food
Book	Non-food
Box	Non-food
Bucket	Non-food
Bus	Non-food
Chair	Non-food
Dock	Non-food
Door	Non-food
Field	Non-food
Forest	Non-food
Flower	Non-food
Forest	Non-food
Road	Non-food
Stair	Non-food
Stapler	Non-food
Waterfall	Non-food

Supplemental Table 2: Buffet Foods

Food	Approximate caloric content (kcal per gram)
Lay's Potato Chips (1 bag)	5.46
Doritos (Nacho Cheese) (1 bag)	4.96
Sun Chips (Garden Salsa) (1 bag)	4.68
Cheetos Crunchy (1 bag)	5.67
Goldfish Crackers (1 bag)	4.74
Skinny Pop Popcorn	5.01
Oreos (4 cookies)	4.72
Famous Amos Minis (1 bag)	5.07
Poptarts (1 pack)	3.91
Ritz Bitz Crackers (1 bag)	5.22
Pretzels (1 bag)	3.95
Hummus (1)	1.74
M&Ms (Plain)	4.92
Skittles	3.84
Chewy Granola Bar (CC) (1)	4.2
Chewy Granola Bar (PB) (1)	4.1
Mini Bagel (Plain) (2)	2.79
Philadelphia Cream Cheese (2 pck)	3.21
Mixed Nuts	6.07
Raisins (2 boxes)	2.99
Apple Slices (1 bag)	0.52
Baby Carrots	0.41
String Cheese (1)	2.55
Greek Yogurt	0.82
Yoplait Yogurt	1.02
**Drinks**	**Approximate caloric content (kcal per gram)**
Coke	0.37
Diet Coke	0.02
Sprite	0.40
Honest Tea (Honey)	0.33
Water	0.00
Vitamin Water	0.21
Starbucks Frappucino (Vanilla)	0.70

Supplemental Table 2. Foods and drinks available at the buffet along with the caloric content of each. Total energy available from the buffet meal was 4650kcal.

SUPPLEMENTAL TABLE 3: ALL PARTICIPANTS BY SUBGROUPS

CHARACTERISTIC	All	Completed Glucose Food-Cue	Completed Water Food-Cue	Completed Glucose Hormones	Completed Water Hormones
SEX	Male: n=32<br>Female: n=40	Male: n=32<br>Female: n=37	Male: n=30<br>Female: n=38	Male: n=29<br>Female: n=37	Male: n=26<br>Female: n=36
AGE (YEARS)	23.22 ± 3.73	23.19 ± 3.77	23.23 ± 3.79	23.17 ± 3.58	23.1 ± 3.58
BMI (KG/M²)	27.33 ± 5.13	27.19 ± 5.1	27.4 ± 5.12	27.29 ± 5.08	27.4 ± 5.22
LEAN	n=25; 22.13 ± 1.67	n=24; 22.03 ± 1.63	n=23; 19.18 ± 1.71	n=22; 22.11 ± 1.68	n=22; 22.3 ± 1.71
OVERWEIGHT	n=25; 26.93 ± 1.28	n=25; 26.93 ± 1.28	n=24; 26.83 ± 1.22	n=25; 26.9 ± 1.28	n=21; 26.85 ± 1.28
OBESE	n=22; 33.68 ± 3.05	n=20; 33.72 ± 3.05	n=21; 33.73 ± 3.12	n=19; 33.75 ± 3.25	n=19; 33.91 ± 3.18
PERCENT CALORIES FROM ADDED SUGAR	9.31 ± 4.47	9.27 ± 4.53	9.47 ± 4.51	9.28 ± 4.49	9.36 ± 4.74
LOW (<10%)	n=42; 6.59 ± 2.38	n=41; 6.54 ± 2.33	n=38; 6.55 ± 2.31	n=40; 6.62 ± 2.2	n=37; 6.48 ± 2.37
HIGH (≥10%)	n=30; 13.16 ± 3.88	n=28; 13.27 ± 3.97	n=30; 13.16 ± 3.88	n=26; 13.37 ± 4	n=25; 13.63 ± 4.09

Supplemental Table 3. Participant characteristics separated by subgroups.

SUPPLEMENTAL TABLE 4A: BMI STATUS

**CHARACTERISTIC**	**Lean**	**Overweight**	**Obese**
SEX	Male: n=10<br>Female: n=15	Male: n=15<br>Female: n=10	Male: n=7<br>Female: n=15
AGE (YEARS)	24.31±4.1	22.16±2.8	23.18±4.02
BMI (KG/M²)	22.13±1.67	26.93±1.28	33.68± 3.05
PERCENT CALORIES FROM ADDED SUGAR	8.46±3.93	10.5±5.51	8.92±3.52
LOW (<10%)	n=14; 5.72±2.72	n=13; 6.79±1.61	n=15; 7.12±2.32
HIGH (≥10%)	n=11; 11.94±1.91	n=12; 14.51±5.45	n=7; 12.77±2.34

SUPPLEMENTAL TABLE 4B: HIGH VS LOW ADDED SUGAR

**CHARACTERISTIC**	**Low Added Sugar (<10%)**	**High Added Sugar (≥10%)**
SEX	Male: n=19<br>Female: n=23	Male: n=13<br>Female: n=17
AGE (YEARS)	23.54±3.96	22.76±3.4
BMI (KG/M²)	27.71±5.54	26.8±4.53
LEAN	n=14; 21.78±1.74	n=11; 22.59±1.55
OVERWEIGHT	n=13; 27.13±1.49	n=12; 26.71±1.04
OBESE	n=15; 33.74±3.28	n=7; 33.55±2.73
PERCENT CALORIES FROM ADDED SUGAR	6.55±2.31	13.16±3.88

Supplemental Table 4. (a) Participant characteristics separated by BMI Status, and (b) Participant characteristics separated by High vs Low Added Sugar.
